# Assessment of cognitive status in patients with type 2 diabetes through the mini-mental status examination: a cross-sectional study

**DOI:** 10.1186/1758-5996-2-10

**Published:** 2010-01-28

**Authors:** Renata C Alencar, Roberta A Cobas, Marília B Gomes

**Affiliations:** 1 Department of Endocrinology and Metabology, Hospital Universitário Pedro Ernesto, Universidade Estadual do Rio de Janeiro (UERJ), Rio de Janeiro, RJ, Brazil

## Abstract

**Background:**

Diabetes is considered an independent risk factor for cognitive impairment and some studies observed through neuropsychological tests that cognitive disfunction affects both elderly and younger patients with diabetes. The aims of this study were to evaluate the cognitive status of outpatients with type 2 diabetes and to evaluate factors associated with impaired function.

**Methods:**

A cross-sectional study was conducted in a group of type 2 diabetic outpatients. They were asked to undergo the Mini-Mental State Examination (MMSE) during routine ambulatory visits between April 2006 and January 2007, with the highest pontuation of the test being 30 points. Patients were classified as having possible dementia according to years of study. Exclusion criteria were blindness, illiterately, stroke, Alzheimer disease and psychiatric disorder. Results are presented as median (interquartile range) or mean ± SD.

**Results:**

The study group was composed of 346 type 2 diabetic outpatients (216 females), aged 58,6 ± 12,1 years and with duration of diabetes of 12,3 ± 9,1 years. Hypertension was present in 77,2%. The total MMSE score achieved was 26 points (16 - 30) and was correlated with years of study (R^2 ^= 0,39, p < 0,001) and 'per capita' income (R^2 ^= 0,22, p < 0,0001) and duration of diabetes (R2 = - 0,13, p = 0,01). Patients who needed help to take their medications obtained worst performance in the MMSE (23,16 ± 3,55 *vs *25,7 ± 2,84, p < 0,01) and were more likely to present possible dementia (p < 0,01). Forty two subjects (12.1%) had diagnosis of possible dementia and this was also associated with years of study (p = 0,045). No association was observed between possible dementia and total MMSE scores with A1C levels.

**Conclusions:**

We conclude that patients with type 2 diabetes should be regularly evaluated for their cognitive function, because duration of disease could be associated with decline in cognition. The early implementation of mini mental which is a simple method of execution can be done to detect early stages of dementia. This test could be an important tool to access the ability of patient to understand their disease and treatment.

## Background

The increasing prevalence of diabetes over the world has become an important public health problem. Diabetes is considered an epidemic disease nowadays, with about 173 million diabetic people over the world. As population is increasing, getting older, more obese and sedentary, the number of individuals with diabetes also increases. The population in Brazil is about 180 million of people [1]. The last national study conducted in 1988 [2] observed a prevalence of diabetes of 7.6%. Recent data in Brazil [3] estimated about 8 million patients with diabetes.

Diabetes is the fifth cause of hospitalization and as an underlying cause of death is among the ten major causes of mortality in our country [4]. According to different studies more than 50% of these patients will die from cardiovascular disease (CVD) [5]. Besides diabetes, these patients usually have other comorbidities, such as hypertension, obesity and dyslipidemia. Today there is strong evidence that supports an intensive control of glycemia, blood pressure, cholesterol and weight to avoid cardiovascular and diabetes chronic complications, aiming to decrease morbidity and mortality of the disease [6,7]. Due to the later events, patients have to change their lifestyle and take many different pills to achieve a good control of all parameters. These factors make it difficult for patients who are not able to conduct theirselves or have low educational and cognitive levels, which may lead to an erroneous interpretation of prescription and, as consequence, a poor adherence to treatment.

Although diabetes is considered to be risk factor for cognitive impairment [8-11] the cognitive function of patients with type 2 diabetes is not usually evaluated in routine clinical care. Cognitive impairment might be another factor associated with poor diabetes control and also with bad adherence of patients to educational approaches, such as diet orientations. Besides this, type 2 diabetes and dementia are common in the elderly [8,9], which are often progressive and disabling conditions. Some studies suggest that chronic hyperglycemia and vascular alterations can cause damage to central nervous system and lead to diabetes-related cognitive impairment [12-14]. Beside this, a recent review showed considerable evidences from the literature on the possible association between type 2 diabetes with Alzheimer's disease [15].

Dementia affects about 8% of all people older than 65 and has become the focus of many studies [16] and its pathophisiology is still unknown. Among the neuropsychological tests, the mini-mental state examination (MMSE) is one of the most widely used screening tests. This test which differentiates patients with non-specified organic brain syndrome and depression from normal individuals is also useful to estimate the severity of cognitive impairment and in documenting serially cognitive changes. The performance in the MMSE can be influenced by age and education level [17-19], especially in developing countries.

The aims of our study were to evaluate the cognitive status of patients with type 2 diabetes and to evaluate factors associated with impaired function detected by MMSE.

## Methods

A cross-sectional study was conducted in a group of 346 type 2 diabetic outpatients attended at the diabetes clinic of the State University of Rio de Janeiro. Patients were considered to have type 2 diabetes when diabetes was diagnosed after 30 years of age, without insulin use in the first year after the diagnosis and without history of ketonuria.

Exclusion criteria were blindness, illiterately, stroke, Alzheimer disease and psychiatric disorders. Patients were asked to undergo the Mini-Mental State Examination (MMSE) during routine ambulatory visits between April 2006 and January 2007.

Demographic characteristics such as sex, age, years of study, 'per capita' income and duration of diabetes, and medical history of hypertension, dyslipidemia, smoking, and treatment of diabetes were assessed.

Hypertension was defined as systolic blood pressure (sBP) ≥140 mmHg and/or diastolic blood pressure (dBP) ≥ 90 mmHg [20] or any value in patients under anti-hypertensive treatment. Dyslipidemia was defined as total cholesterol ≥ 200 mg/dl and/or HDL ≤ 45 mg/dl and/or LDL ≥ 100 mg/dl and/or triglycerides ≥ 150 mg/dl and/or use of statins or fibrates.

Smoker and former smoker were considered those who make or have made use of any amount of cigarettes, respectively and were considered together in a group of ever smokers.

Patients who need help to take their medications were considered those who need a second person to administer medications as prescribed.

Glycated Haemoglobin (A1c) was determined using a high performance liquid chromatography (HPLC) reference value of 4 - 6.2%. FBG, triglyceride, HDL cholesterol and total cholesterol levels were measured using an auto-analyzer (Cobas-Mira Roche) with enzymatic techniques. LDL cholesterol was calculated according to Friedewald when triglycerides levels were less than 400 mg/Dl [21]. Weight and height were measured to the nearest 0.1 Kg and 0.1 cm respectively. Body mass index (BMI) (Kg/m^2^) was calculated using these measurements.

A standard MMSE form (22) was administered to each of the 346 subjects. The MMSE scale ranges from 0 to 30 points, with higher number indicating better performance. Instructions were identical for each subject.

The MMSE consists in 19 questions designed to assess the patient's mental status [21] in the following 5 categories: 10 orientations questions (year, season, date, day, month, state, city, close street, floor and location); 2 memories items (repeat the words car, window and vase and after delayed recall); 1 calculation item; 5 language items (naming 'watch-pencil', repeat 3 words 'No ifs, ands, or buts' (adapted to Portuguese language as 'nem aqui, nem ali, nem lá'), 3 step-command, read and follow the sentence 'close your eyes' and write a sentence) and 1 constructional item (copy overlapping pentagon) (Appendix 1).

The stratification of the cut-off points was done in accordance to years of study in order to prevent the possibility of education level to mask the performance in this test. We used a score system validated on Brazilian population [22], with the following interpretation to possible dementia: MMSE score < 26, if patient had more than 8 years of study; MMSE score < 18 if patient had between 1 and 8 years of study.

### Statistical analysis

The Kruskal-Wallis and Mann-Whitney U tests were used for comparisons between groups of variables not normally distributed and the Student t-test and ANOVA were used for the other comparisons. ANCOVA was used for controlling for the following variables (age, gender, duration of diabetes, years of study, hypertension, dyslipidemia, HbA1c). The Chi-square test with Yates correction was used for comparison of categorical variables. Pearson correlation and partial correlation controlling for the following variables (age, gender, duration of diabetes, years of study, hypertension, dyslipidemia, HbA1c) using Pearson's correlation coefficient was also performed.

These analyses were performed using the statistical package for the social science (SPSS, version 13.0). Values were expressed as mean (SD) or median (interquartile range). A two-sided P value less than 0.05 was considered to be significant.

## Results

The study group was composed of 346 type 2 diabetic outpatients (130 males, 216 females), aged 58.6 ± 12.1 years and with duration of diabetes of 12.3 ± 9.1 years. Demographics and clinical characteristics of the patients are shown in table 1. Fifty nine percent of patients had between 8 or less years of study and 41% had more than 8 years.

**Table 1:** Demographic charactheristics of the patients

Total (n)	346
Female (n/%)	216/62.4%
Male (n/%)	130/37.6%
Age (years)	58,6 ± 12,1
Duration of diabetes (years)	12,3 ± 9,1
Years of study	7.58 ± 4.07
HbA1c (%)	7.92 ± 1.83
Hypertension (%)	77.2%
Dyslipidemia (%)	76.9%
Smokers (%)	9.2%

Diet alone and one or more Oral Hypoglycemic Agents (OHA) were the treatment of respectively 2% and 56% of the diabetic patients. Insulin therapy alone was seen in 19% and insulin plus one or more OHA in 23% of the patients studied. Most patients used 4 or more drugs for the treatment for diabetes and comorbidities, corresponding to 71.8% of the sample. We did not observe association between total number of drugs used and glycemic control measured by A1c neither with MMSE score.

The mean time spent on performing the MMSE test was 16 minutes.

The mean total MMSE score was 26 points, with a range from 16 to 30. Men presented higher MMSE score than women (26.3 ± 3.2 *vs *24.8 ± 2.7; p < 0.01), and more years of study (9.2 ± 5.7 vs 7.2 ± 3.5; p = 0.001). The association between MMSE and gender persisted after controlling for age (p = 0.001), HbA1c (p = 0.001), hypertension (0.001), dyslipidemia (p = 0.001) and duration of diabetes (p = 0.001) In this model gender and years of study have the same weight.

Patients who needed help to take their medications obtained worst performance in the MMSE (23.16 ± 3.55 *vs *25.7 ± 2.84, p < 0.0001) which persisted after controlling for age, gender, duration of diabetes, years of study, hypertension, dyslipidemia, and HbA1c (p < 0.001 for all). These patients were more likely to present possible dementia (p < 0.01).

Diagnosis of hypertension and dyslipidemia were both associated with total MMSE score (25.16 ± 3.05 *vs *26.08 ± 2.91, p = 0.014 and 25.16 ± 3.06 *vs *26.08 ± 2.91 p = 0.014, respectively). The association with hypertension persisted after controlling for age (p = 0.03), but not after controlling for duration of diabetes (p = 0.07) and years of study (p = 0.14). The association with dyslipidemia, persisted after controlling for age (p = 0.04) and years of study, but not after controlling for duration of diabetes (p = 0.055). The small number of patients with dementia (n = 13) did not allow further analysis. There was no association between smoking and score test.

No association was observed between possible dementia and total MMSE scores with A1C levels.

A correlation was found between duration of the diabetes and the amount of points obtained in MMSE (r = - 0.13; p = 0.01), that did not persist after controlling for years of study (r = -0.08 p = 0.12), Further analysis showed that the correlation between duration of diabetes and MMSE persisted after controlling for HbA1c (r = - 0.14 p = 0.01), age (r = -0.13 p = 0.02), gender (r = -0.11 p = 0.03), hypertension (r = -0.13 p = 0.01) and dyslipidemia (r = -0.14 p = 0.01). The diagnosis of possible dementia was not associated with duration of the disease.

The mean total MMSE score was correlated with number of years of study (r = 0.39, p < 0.001). Forty two subjects (12.1%) had diagnosis of possible dementia and this was also associated with years of study (p = 0.045).

In our sample, number of years of study was correlated with age (r = - 0.11; p = 0.03). There was no correlation between age and score test. No quantitative difference in score test was found between patients with less (n = 279) or more (n = 65) than 70 years of age but a qualitative difference was found. Patients older than 70 years had lower score when evaluated for 'spatial orientation' (p = 0.034), 'evocation of memory' (p = 0.007) and 'praxia' (p = 0.038) compared to younger than 70 years.

The 'per capita' income was less than $290.00 in 75% of patients. A correlation was found between 'per capita' income and score test (r = 0.22; p < 0.0001).

## Discussion

Diabetes is a chronic disease that leads to chronic long term complications, including risk of cognitive changes. In order to achieve intensive metabolic and blood pressure control it is quite often necessary a complex prescription, with several number of drugs. In this way, a preserved cognitive status is essential to the understanding of treatment and compliance.

In our study, we screened the mental state of patients with type 2 diabetes through the mini mental status examination.

MMSE may suffer influence of schooling [23,24], which is of particular interest in developing countries. According to this in our sample the total score achieved in the test was greater in patients with higher education level and patients classified as having possible dementia had fewer years of study. Men had better performance in the test and also had more years of study than women.

Patients with longer duration of diabetes achieved lower score test in agreement with other series [25,26]. Similar to other study [25] glycemic control measured by A1C had no association with MMSE score and possible dementia in the evaluation period. It is important to mention that we did not consider previous A1C values, but only the A1C that has measured in the same year of MMSE test. The above-mentioned fact did not permit us to analyse glycemic control throughout the duration of the disease. In contrast Reaven *et al*. demonstrated correlation between several measures of cognitive functions and degree of hyperglycemia [27]. In this study, with the same sample, they showed that the reduction in A1C levels was associated to better cognitive performance in elderly patients with type 2 diabetes [28].

The total number of drugs used by patients had no association with glycemic control. This finding may indicate that despite successive combinations of drugs aiming to achieve good metabolic control it is important to assess adherence to treatment. The ability of the patient to understand the prescription and remember the information provided during the appointment are important to increase compliance to treatment. In addition, patients with possible dementia and lower scores related need for assistance to take their medication. This indicates the importance of greater attention to these patients because cognitive deterioration leads to impairment in self-management ability [29]. Despite we did not find difference in the total MMSE score, our patients older than 70 years presented worse performance concerning memory evocation, which may represent difficulty in incorporating information provided during a medical consultation.

Diabetes usually does not occur isolated. Hassing et al. demonstrated that hypertension alone was not associated to cognitive decline, but when combined to diabetes it increases the risk of cognitive impairment [30] which is consistent to prior studies [31,32]. In our study, patients with hypertension and dyslipidemia had lower scores in the test, and it is known that these co morbidities are related to atherosclerosis.

In our study, smoking was not associated to the punctuation achieved on MMSE test. In contrast Arvanitakis et al. found strong association of diabetes with cognitive impairment in current smokers compared to those who never smoked or formerly smoked.

Of the 346 patients who underwent the test, 42 (12.1%) were classified as presenting possible dementia. Although our patients are younger than patients in other studies and also young to assess possible diagnosis of dementia, we found prevalence similar to others studies with older people [25,33]. This finding suggests that diabetes could be a more important risk factor than age in the development of cognitive dysfunction, even though our study did not evaluate a control group to assess the real impact of diabetes in cognitive function. As above mentioned other factors, such as hypertension and dyslipidemia may have influenced our results. However it is important to emphasize that both age of the subjects and criteria used for diagnosis of possible dementia are different between our study and the studies mentioned above which makes it difficult the comparison among them.

Much of our population lives in poor socioeconomic conditions and a large contingent of Brazilians are illiterate or have low educational level. 'Per capita' income can reflect socioeconomic status and in our country people with better income have better educational level. This might be the reason that in our sample lower 'per capita' income has been linked to lower scores.

Our study has also some limitations. It's known that physical activity has a significant impact on cognitive function, with a study showing probable mediation of insulin-related systems in the brain and skeletal muscles [15]. First, we did not evaluate exercise and alcohol history. The investigation of some other factors including smoking and years of study was based on self-report which may have lead to recall bias. Second, as MMSE is considered a screening test to evaluate dementia, we did not use other methods to better evaluate or to confirm this possible diagnosis. These tests such as magnetic resonance are expensive and difficult to be used in routine clinical care. Third, given the socioeconomic status of Brazilian patients, years of study do not necessarily reflect a proper schooling.

## Conclusions

Patients with type 2 diabetes should be regularly evaluated for their cognitive function, because duration of disease could be associated with decline in cognition. Based on the definition of health that is the wellness of body, mind and social status; the maintenance of the cognition should be chased as the protection of other target-organs usually affected by diabetes, and are normally part of medical routine care. The early implementation of mini-mental, which is a simple method of execution, can be done to detect early stages of dementia. This test could be an important tool to access the ability of patient to understand their disease and treatment. Future studies will be important to better identify risk factors for cognitive dysfunction and lighten its relationship with diabetes.

## Competing interests

The authors declare that they have no competing interests.

## Authors' contributions

RCA participated in acquisition of data and drafting the manuscript

RAC participated in drafting and revising the manuscript, performed statistical analysis

MBG participated in study design, drafting and revising the manuscript, performed statistical analysis and gave final approval of the version to be published

All authors read and approved the final manuscript.

## Appendix 1 - Mini-mental State Examination

10 orientations questions (year, season, date, Day, month, state, country, city, floor and location) - 1 point for each right question

1 memory item (delayed recall of car, pot, penny) - 1 point for each right question

1 item for Calculation: subtraction of seven serially (100-7; 93-7; 86-7; 79-7; 72-7; 65)- 1 point for each correct calculation

6 language itens:

"watch-pencil" [naming] - 1 point each registration of 3 words (car, pot, penny) - 1 point each registration of "No ifs, ands, or but" [repetition] - 1 point

3-step command [comprehension] - 1 point for each step

"close your eyes" [reading] - 1 point

"write a sentence" [writing] - 1 point

1 constructional item (copy overlapping pentagons) - 1 point
